# Physical activity as a modifiable risk factor for periodontal disease

**DOI:** 10.3389/froh.2023.1266462

**Published:** 2023-11-13

**Authors:** Charlotte Cheuk Kwan Chan, Alice Kit Ying Chan, Chun Hung Chu, Yiu Cheung Tsang

**Affiliations:** Faculty of Dentistry, The University of Hong Kong, Hong Kong, Hong Kong SAR, China

**Keywords:** physical activity, periodontitis, modifiable risk factor, periodontal disease, elite athlete, general public

## Abstract

Non-communicable diseases (NCDs), which contribute significantly to global morbidity, are largely preventable through behavioral changes. As with other NCDs, periodontitis is associated with modifiable risk factors such as smoking and stress and is linked to multiple adverse health outcomes through a shared pathway of chronic systemic inflammation. While the health benefits of physical activity have been widely promoted in public health and extensively studied for other systemic conditions, its impact on periodontal health has only recently started to gain attention. This article critically evaluates the current literature on the relationship between physical activity and periodontitis. While cross-sectional studies have shown an inverse association between physical activity levels and periodontitis risk in the general population, clinical oral health surveys of elite athletes with high levels of physical activity have nonetheless revealed poor periodontal conditions. Although causality has not been determined, physical activity could positively impact periodontitis directly, by reducing inflammatory biomarkers, and indirectly, through its modulatory effects on insulin sensitivity, obesity, bone density, stress, and other health promoting behaviors. Given the importance of risk factor control during initial periodontal therapy, understanding the role of physical activity as a potential behavioral risk modifier is paramount. The findings of this review provide an evidence-based overview of how physical activity could influence periodontitis. There is a need for longitudinal cohort studies to verify the temporality of the reported associations and exclude confounders, while interventions are needed to assess the efficacy of physical activity on periodontal treatment outcomes.

## 1. Introduction

Physical activity has been widely promoted as a public health measure in the prevention and management of non-communicable diseases (NCDs) (1–3). Evidence supports the beneficial role of physical activity in reducing stress, adipose tissue and systemic inflammation (4), as well as improving circulation, insulin sensitivity and musculoskeletal health (5). For this reason, physical activity is listed by the World Health Organization (WHO) as one of the four modifiable behavioral risk factors in NCD prevention, together with diet, tobacco and alcohol use (6). Although the WHO recommends adults aged 18-64 to complete at least 150 min of moderate-intensity or 75 min of vigorous-intensity aerobic physical activity weekly, more than a quarter of adults do not meet this target (7). At the same time, there has been a rapid rise in the number of NCDs worldwide, which account for nearly three-quarters of overall deaths despite being largely preventable (6). Physical inactivity is now the fourth leading risk factor for global mortality (8) and is estimated to be responsible for up to 10% of NCDs (9), including diabetes, heart disease and certain cancers, creating significant economic and societal burdens.

Periodontal disease is a preventable chronic disease that affects up to half of the global population and shares common risk factors with other NCDs (10). As with the majority of NCDs, inflammation underpins the progression of periodontal disease, which is characterized by an excessive inflammatory host response to microbial dysbiosis resulting in destruction of the supporting structures of the dentition. Tooth loss attributed to periodontitis profoundly reduces individual quality of life through impairments in speech, mastication and psychosocial wellbeing, resulting in a global estimated cost of US$54 billion in lost productivity per annum (11).

Behavioral modification, effective and sustained dental biofilm control, and the management of risk factors are considered primary components of periodontal disease prevention and therapy (12). While current oral health interventions have focused on tobacco cessation (13), oral hygiene instruction (14, 15), and dietary modification (16), physical activity has not been widely studied as a risk indicator for periodontitis. However, emerging evidence suggests that physical activity is associated with improved periodontal outcomes (17–19). Guidance from the Scottish Dental Clinical Effectiveness Programme on the Prevention and Treatment of Periodontal Diseases in Primary Care recommends discussions with patients regarding the benefits of regular exercise (20). The European Federation of Periodontology's S3 Clinical Practice Guidelines acknowledge the need for additional research to assess whether physical activity positively impacts periodontal treatment outcomes, but nonetheless include it as a potential risk indicator to be controlled in the first step of periodontitis therapy (12).

The aim of the present review is to summarize the available evidence on the relationship between physical activity and periodontal disease. Many of the studies that demonstrate an association between activity levels and periodontal disease severity are limited by their cross-sectional design, the influence of confounders, and differences in the measurement and definition of physical activity. It has also been found, paradoxically, that elite athletes have poor periodontal conditions despite good oral hygiene practices (21–23). Understanding the role of physical activity in periodontitis risk will not only help dentists deliver more comprehensive behavioral change interventions for the prevention and management of this multifactorial disease, but may influence future health promotion guidelines for both oral and general health.

## 2. Literature search

To prepare this narrative review, a literature search was conducted with the keywords [(exercise) OR (physical activity) AND (periodontitis) OR (periodontal disease)] in PubMed, Web of Science, and Google Scholar. Only articles from 2005 to 2023 published in English with full-text availability and clinical periodontal outcomes were included. After duplicate removal, titles and abstracts were screened. Studies were rejected if physical activity was not one of the main variables analysed, but only included to reduce the influence of external variables in measuring associations of periodontitis and other conditions, such hypertension (24) or dementia (25). The full texts of the remaining papers were reviewed, resulting in the inclusion of 12 studies investigating the association between physical activity and periodontal disease and 11 studies investigating the periodontal conditions of elite athletes.

## 3. Physical activity levels and periodontal disease

A recent meta-analysis found that regular physical activity reduced periodontitis risk by 23% (26). Physical activity has been defined by the WHO as “any bodily movement produced by skeletal muscles that requires energy expenditure” (27). An internationally validated measurement tool used to assess levels of physical activity is the International Physical Activity Questionnaire (IPAQ), a self-report questionnaire that measures frequency and duration of different intensities of physical activities across the last 7 days. Despite the recall bias associated with self-report measures, the use of the IPAQ with its clear scoring protocol enables greater standardization when describing physical activity levels across different adult populations. It also facilitates comparisons across studies, as the results from the IPAQ can be categorically scored as low, moderate or high physical activity levels based on preset definitions, or calculated into continuous data in the form of MET-minutes, which represent multiples of resting metabolic rate. The physical activities surveyed are recorded under different domains, such as leisure-time, occupational and transport-related physical activity, thus enabling the undertaking of more specific analysis. The Global Physical Activity Questionnaire developed by the WHO has also been used to survey physical activity levels across a typical week and has shown reliability and correlation with IPAQ (28).

Across all eight of the included cross-sectional studies, lower levels of physical activity were associated with greater prevalence of periodontal disease. Four studies used the IPAQ to study the existence of an independent association between periodontitis severity and levels of physical activity in adults from Italy (18), Jordan (19) and South Korea (29, 30). Models were adjusted for covariates that could affect periodontitis such as smoking, diabetes mellitus, age, gender, occupation, education level, household income, and BMI, as shown in Table 1. High levels of physical activity were significantly associated with a lower prevalence of stage III and IV periodontitis (18), lower gingival inflammation, probing depths (30) and a reduced number of sites with clinical attachment loss ≥3 mm (19). Three studies also investigated the role of dietary practices and found that the combination of a healthy diet and regular exercise habit reduced the odds of severe periodontitis by 10 times (18). Rather than investigate physical activity in general, Han et al. used a modified version of IPAQ to specifically look at the impact of regular walking practices. The study revealed that regular walking significantly lowered the risk of periodontal disease, suggesting that even low-intensity physical activity could be of benefit in reducing periodontitis risk. Moreover, physical activity attenuated the relationship between periodontitis and low socioeconomic status, as periodontitis was significantly associated with low socioeconomic status in non-regular walkers, but not in the regular walking group. This finding could be important in tackling social inequalities in oral health (29).

**Table 1:** Odds of Periodontal Disease with Different Levels of Physical Activity (PA).

Study (Ref)	OR (95% CI)	PA parameter	PD definition	Covariates adjusted for in OR calculation						
				Sex	Age	Smoking	DM	Edu	Income	Other
Inadequate or Low Level of PA										
Marruganti et al., 2022 (18)	1.65 (0.84, 3.28)	Low level of PA (IPAQ)	Stage III/IV based on 2017 World Workshop Case Definitions	✓	✓	✓				Bushing frequency
Bawadi et al., 2010 (19)	3.8 (1.6, 9.0)	Low level of PA (IPAQ)	≥4 teeth with PPD ≥4 mm and CAL ≥3 mm	✓	✓		✓	✓		BMI, missing teeth, plaque index
Hwang et al., 2022 (30)	1.16 (1.04, 1.30)	Inadequate PA (IPAQ)	CPI 3 or 4	✓	✓	✓	✓		✓	Alcohol
Marruganti et al., 2023 (28)	1.47 (1.26–1.72)	Low leisure time PA and high occupational PA	AAP/CDC definition	✓	✓	✓		✓	✓	
Almohamad et al., 2022 (17)	1.17 (1.00–1.36)	Sedentary behavior >7.5h per day (GPAQ)	AAP/CDC definition	✓	✓	✓	✓	✓	✓	
Almohamad et al., 2022 (17)	1.13 (0.83–1.54)	No leisure time moderate-vigorous PA	AAP/CDC definition	✓	✓	✓	✓	✓	✓	
Regular or High Level of PA										
Han et al., 2019 (29)	0.793 (0.699–0.898)	Regular walking (IPAQ)	CPI 3 or 4	✓	✓	✓	✓	✓	✓	
Marruganti et al., 2023 (28)	0.81 (0.72–0.92)	High leisure time PA (GPAQ)	AAP/CDC definition	✓	✓	✓				
Al-Zahrani et al., 2005 (31)	0.58 (0.35–0.96)	Meeting WHO recommended level of PA (MET)	≥1 site with PPD ≥4 mm and CAL ≥3 mm	✓	✓	✓		✓	✓	BMI, Race, diet, dental visit
Iwasaki et al., 2023 (25)	0.64(0.46–0.89)	Top quintile of total PA vs bottom quintile (MET)	AAP/CDC definition	✓	✓	✓	✓	✓	✓	BMI, interdental cleaning, dental visit, brushing frequency

OR, Odds ratio; PA, Physical activity; PD, Periodontal disease; DM, Diabetes mellitus; Edu, Education level; CPI, Community Periodontal Index; CAL, Clinical attachment loss; IPAQ, International Physical Activity Questionnaire; GPAQ, Global Physical Activity Questionnaire; MET, Metabolic Equivalents.

Two studies used the GPAQ to assess levels of physical activity in different domains, namely transportation, occupation and leisure time (17, 28). The latter refers to bodily movements “performed during free time”, “at the subject's discretion”, and “not required as part of the essential activities of daily living”, while occupational physical activity is differentiated by referring to bodily movements contributing towards the subject's professional duties and includes “carrying/lifting heavy loads, digging or construction work, household chores, etc.” Interestingly, while higher leisure time physical activity was a protective indicator for periodontitis, there was a divergent relationship when occupational physical activity was considered, with greater levels of physical activity at work leading to an increased periodontitis risk (28).

In a large-scale population study of US adults, a significant association was found between increased physical activity and lower periodontitis prevalence in never and former smokers (31). However, while former smokers who met the recommended levels of physical activity were 74% less likely to have periodontitis compared to those who were inactive, this relationship was not significant for current smokers, suggesting that the effect of smoking may override any potential periodontal health benefit from exercise.

## 4. Mechanisms for the relationship between physical activity and periodontitis

**Figure 1:**
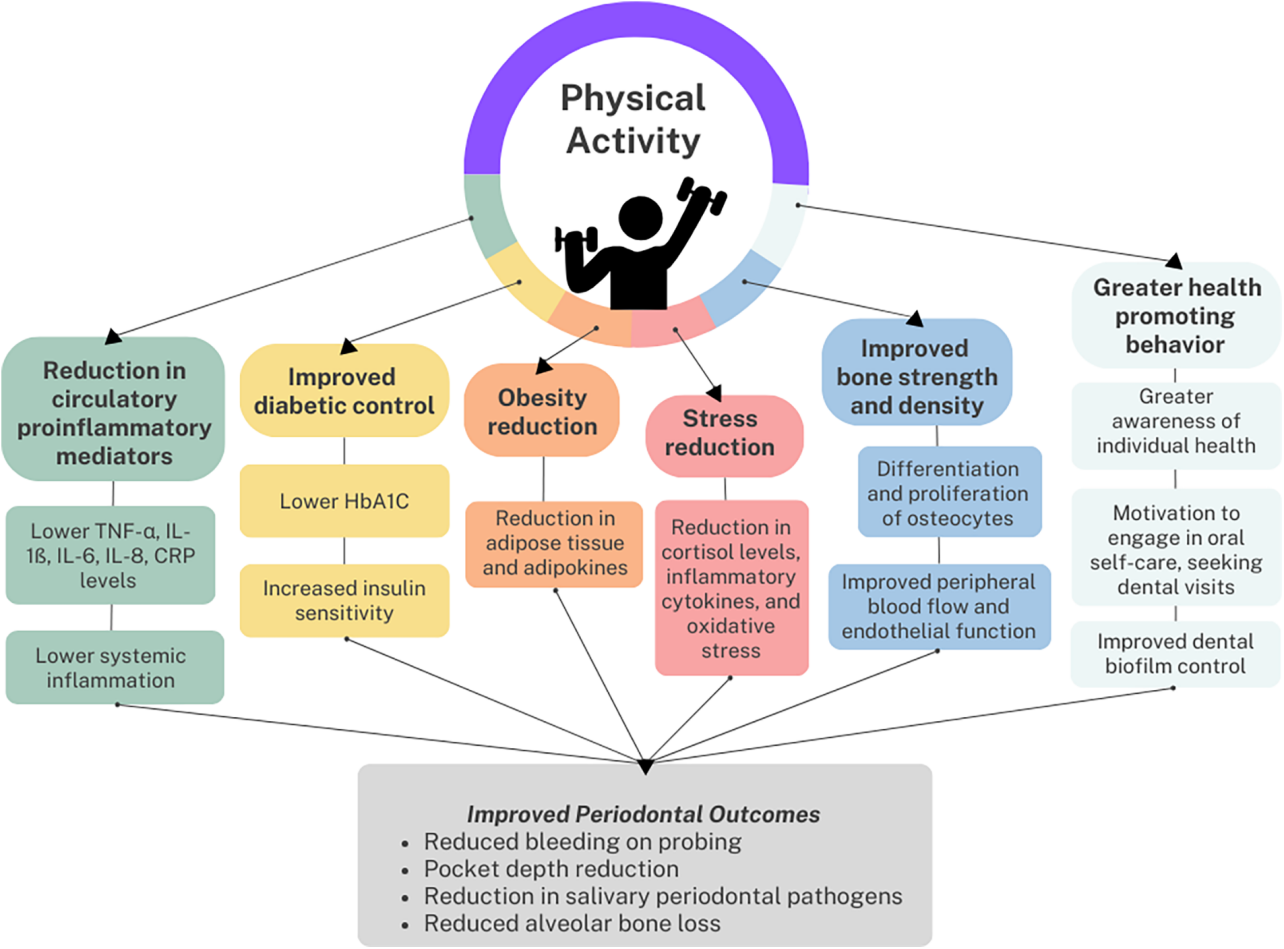
Reported association and the respective potential links between physical activity and periodontal health.

Several underlying mechanisms have been proposed to explain the association between periodontitis and physical activity, which are summarized in Figure 1. Physical activity may directly lower the risk of periodontitis by reducing the level of circulatory proinflammatory mediators associated with periodontal connective tissue attachment loss and alveolar bone resorption (32). A case control study of 751 Australian adults found that those engaging in the recommended amount of leisure time physical activity had lower odds of elevated interleukin-1ß and C-reactive protein (CRP) in gingival crevicular fluid compared to those who were insufficiently active (33). In addition, a dose-response relation was found among periodontitis cases, with increasing levels of physical activity resulting in decreased probability of detectable CRP. Other studies have found that physical activity lowered serum TNF-α, IL-6 and IL-8 levels (25, 32, 34), which could promote periodontal health. Apart from its anti-inflammatory effect, physical activity could suppress periodontal destruction by improving peripheral blood flow and endothelial function (25, 35).

Physical activity could also indirectly protect against periodontitis by reducing known periodontal risk factors, such as diabetes mellitus (36). Physical activity lowers glycated hemoglobin (HbA1C) levels and increases insulin sensitivity, resulting in improved diabetic control (37, 38). While most cross-sectional studies showing an association between physical activity and periodontal disease have excluded diabetes mellitus as a potential confounder, a recent randomized controlled trial was conducted to assess the effect of a 6-month physical activity intervention on the periodontal health of type 2 diabetes mellitus patients. Compared to the control group, those who received the intervention were found to have a significant reduction in bleeding on probing, periodontitis severity, and HbA1C levels (39).

In addition, obesity (40, 41) and intra-abdominal fat (42) have been associated with periodontitis through shared pathways of chronic low-grade inflammation and immune cell dysregulation. Cross-sectional epidemiological studies show that periodontitis is inversely associated with BMI (35) and waist circumference (40). An intervention study among obese men in Japan found a significant decrease in the number of pockets ≥4 mm, the number of teeth with bleeding on probing, and salivary counts of periodontal pathogens including *Tannerella forsythia* and *Treponema denticola* (43) after a 12-week exercise program compared to baseline measurements*.* The study also determined a positive correlation between levels of periodontal pathogens, body weight and fasting insulin, providing further grounds for a mechanism of a shared inflammatory pathway between physical activity, periodontitis and metabolic disorders (32). Physical activity combats obesity by increasing energy expenditure and reducing adipose tissue mass (32), lowering the level of adipokines, macrophages and systemic inflammation (44) which may also benefit periodontal health. While obesity disrupts bone homeostasis by favouring bone resorption which could lead to greater alveolar bone loss (41), the repeated mechanical load and muscular contraction of physical activity promote the differentiation and proliferation of osteocytes. This leads to increased bone strength and density (45), factors which have been associated with less alveolar bone loss (46).

Another potential mechanism is through the stress-reducing effect of physical activity. Randomized controlled trials have demonstrated the positive effects of physical activity on mental health outcomes and a study of over a million American adults found that those who regularly engaged in physical activity reported greater emotional wellbeing than those who were inactive (47). This may be clinically significant for periodontitis patients, as community studies have found psychosocial stress to be associated with greater clinical attachment loss even after adjusting for sociodemographic factors, smoking and diabetes mellitus (48, 49). In a 12-week clinical trial, participants in the intervention group participated in a yoga-based program on top of standard periodontal treatment, while those in the control group only received the periodontal treatment. The intervention group reported reduced stress and had significantly lower plaque scores, bleeding on probing, probing depths and clinical attachment loss upon clinical examination (50). The role of stress is also highlighted in the study by Marruganti et al. (28), who differentiated between leisure-time and occupational physical activity. High levels of occupational physical activity were significantly associated with a greater risk of severe periodontitis, which further increased when combined with low leisure-time physical activity. This was explained through the increased stress experienced when heavy physical labor forms part of one's work, which may result in the activation of the hypothalamic-pituitary-adrenal axis and higher levels of cortisol, pro-inflammatory cytokines and oxidative stress. Conversely, in leisure-time physical activity, psychosocial stress and its associated biological effects are reduced, hence the reverse association is seen with periodontitis.

Finally, it has been proposed that the association could be due to greater awareness and prioritization of health promoting behaviors (31). Individuals who engage in more regular physical activity may have greater motivation to engage in consistent oral hygiene practices or seek regular dental care. This may result in improved periodontal condition given the importance of effective biofilm control in the prevention of a large proportion of periodontal diseases (12). However, behavioral change is unlikely to be the sole explanation as some of the studies demonstrating an association between physical activity and periodontal disease had already excluded oral hygiene habits such as toothbrushing frequency and plaque control in the analysis (18, 19).

## 5. Periodontal conditions of elite athletes

Given the association between periodontal disease and physical activity, it might be expected that elite athletes, with their active lifestyle and strong emphasis on optimal physical condition, would have high levels of periodontal health. However, cross-sectional studies of this population have nonetheless found evidence of periodontal disease (21–23, 51–54). Gingival bleeding on probing or calculus was found in over three-quarters of UK elite athletes (22), gingivitis in 64% of Dutch athletes (53), and periodontitis in 41% of professional football players (51). Periodontal pocket depths of ≥4 mm were found in 27% of elite athletes from Pakistan (21), 15% of athletes presenting at the dental clinic at the London 2012 Olympic Games (55) and 34% of athletes at the Lima 2019 Pan American Games (52). Furthermore, athletes may have a higher incidence of gingival hypertrophy resulting from anabolic steroid use and “swimmers’ calculus”, a build-up of calculus in frequent swimmers, may arise from prolonged exposure to chlorinated water (56).

These studies are limited by their small sample size and the heterogeneity in both the periodontal measurements and nature of sports represented by the athletes. Additionally, most studies did not provide data to compare the prevalence of periodontal diseases observed in the athletes to a control group or population of a similar age and background, limiting the significance of the findings (51, 53). Gallagher et al. found that 22% of UK elite athletes had pocket depths of ≥4 mm which is only slightly higher than the 19% obtained from England's national oral health survey data of a similar age range (22). Gingivitis has been found to affect almost all adults (57) but data specific to the mid-twenties age range of the athletes of the included studies is scarce. Nonetheless, these results are worrying given the relatively young age of the athletes and the tendency for periodontal disease to become more severe with age due to cumulative damage over time. Moreover, the athletes' oral health problems were discovered to negatively impact their daily activities and training performance (21, 22, 55). Further research could potentially reveal whether underlying biological mechanisms due to the physiology of physical activity or stress place them at greater risk of periodontal disease. With sport encompassing the chosen profession of many professional athletes, this may be all the more likely in light of the data showing the association between occupational physical activity and more severe periodontal disease (28).

Based on the inflammatory nature of periodontitis, it has been proposed that athletes could be prone to periodontal problems due to the immunomodulatory effects of strenuous exercise regimes. While individuals engaging in regular moderate physical activity have improved immune function compared to those with a sedentary lifestyle, prolonged intense physical activity has been associated with a short-term depression of immune function up to 24 h after exercise (58), resulting in an “open window” of increased susceptibility to infection. A systematic review of inflammatory biomarkers in high and moderate intensity physical activity revealed that the former could lead to significantly higher levels of inflammatory mediators, especially IL-6, due to muscle damage during the physical activity (59). The review also suggested that intense physical activity could impair immune response if not accompanied by adequate rest (60). Changes in saliva have also been attributed to poorer periodontal conditions in athletes. During exercise, mouth breathing and fluid loss can result in reduced salivary flow and dry mouth (23, 61), which could create a more favorable environment for periodontal pathogens. Salivary cortisol levels, which have been associated with more severe periodontitis (62), were significantly increased after physical activity of high intensity but not low intensity (63). From a behavioral perspective, athletes may place greater focus on improving physical fitness and ignore other aspects of health including oral care. Periodontal disease could arise due to poor compliance with dental appointments and oral hygiene instruction (64), as well as frequent consumption of carbohydrate-rich sports drinks which have been associated with higher BPE scores (21). Finally, as several of the studies on elite athletes' periodontal conditions collected data in the lead up to or during high profile athletic events such as the Olympics (53, 55) and regional championships (52), the impact of psychological stress in the athletes surveyed should be considered when interpreting these results.

## 6. Summary

Emerging evidence of the association between physical activity and periodontal disease may hold promise that exercise interventions, which are already being used to promote general health and wellbeing, could also provide clinical benefit in the prevention and management of periodontal diseases.

Studies have demonstrated an association between increased physical activity and reduced periodontal disease severity through clinical measures such as clinical attachment loss, probing depths, and bleeding on probing. The mechanisms underlying this association have been explained through the effect of physical activity in reducing systemic inflammation and shared risk factors such as diabetes mellitus, obesity and stress. There is evidence that even low intensity physical activity such as walking could reduce periodontal risk (29). On the other hand, prolonged high intensity physical activity could result in greater periodontal risk due to higher levels of inflammatory mediators associated with muscle damage and repair. While studies have highlighted the prevalence of periodontal diseases in elite athletes, the small sample size and lack of population data and a control group create uncertainty as to the clinical significance of such findings.

This review is subject to several limitations, including selection bias arising from the broad literature search conducted. Since the current evidence is limited and largely cross-sectional, causation cannot be inferred. As was previously conducted for smoking cessation and diabetes mellitus management, randomized controlled trials are needed to assess the impact of exercise-based interventions on periodontal treatment outcomes. Moreover, the existing available research focuses largely on data from population surveys. There is a need for laboratory and animal-based studies focusing on the processes underlying the observed associations to derive mechanistic insights into how the host-microbiome interaction might be affected by physical activity.

Another challenge of the present topic is the heterogeneity in the measurement of physical activity and periodontal disease. Physical activity is a broad construct complicated by its ubiquity in daily life activities including leisure, transport and work. The nature of exercise can also vary significantly, having been described with differing and ill-defined terminology such as aerobic, endurance, strength, flexibility or resistance-based activities. While the impact of variations in the definition of physical activity is minimized as the majority of the included studies used standardized measurement tools such as the IPAQ or GPAQ, these measures rely on self-report measures of physical activity, which may be subject to bias and inaccuracy. Future intervention studies should include standardized methodological variables to quantify and determine the intensity, types, volume and frequency of physical activity, especially given the findings that show divergent outcomes when physical activity is separately analysed under the domains of occupation and leisure-time activities. More recent studies have defined periodontitis using the updated 2017 World Workshop periodontitis case definition, but earlier studies relied on simple epidemiological measures such as BPE which may underestimate periodontal disease.

Finally, while the studies included in this review have attempted to control for variables that could affect the relationship between exercise and periodontal disease, it is acknowledged that there may be additional unaccounted confounders. Longitudinal cohort studies may shed light on these confounders and establish temporality. The interrelationship of confounding factors such as stress further complicates the investigation of the mechanisms underlying how physical activity affects periodontal disease.

With greater awareness of the socioeconomic cost of non-communicable diseases as well as the role of lifestyle habits in their prevention, recent consensus reports in dentistry have emphasised behavioral change and risk factor control as first steps in the management of oral diseases (12). Knowledge on the role, if any, that exercise plays in periodontal therapy would enable the development of evidence-based clinical practice guidelines and provide dental practitioners with key information to motivate patients to improve both their oral and systemic health.
